# A Novel LC–MS/MS-Based Method for the Diagnosis of ADA2 Deficiency from Dried Plasma Spot

**DOI:** 10.3390/molecules26185707

**Published:** 2021-09-21

**Authors:** Alessia Cafaro, Federica Pigliasco, Sebastiano Barco, Federica Penco, Francesca Schena, Roberta Caorsi, Stefano Volpi, Gino Tripodi, Marco Gattorno, Giuliana Cangemi

**Affiliations:** 1 Chromatography and Mass Spectrometry Section, Central Laboratory of Analysis, IRCCS Istituto Giannina Gaslini, 16147 Genoa, Italy; alessiacafaro@gaslini.org (A.C.); federicapigliasco@gaslini.org (F.P.); GinoTripodi@gaslini.org (G.T.); giulianacangemi@gaslini.org (G.C.); 2 Center for Autoinflammatory Diseases and Immunodeficiencies, IRCCS Istituto Giannina Gaslini, 16147 Genoa, Italy; federica.penco@hotmail.it (F.P.); francescaschena@gaslini.org (F.S.); robertacaorsi@gaslini.org (R.C.); stefanovolpi@gaslini.org (S.V.); marcogattorno@gaslini.org (M.G.); 3 Clinics of Pediatrics and Rheumatology, IRCCS Istituto Giannina Gaslini, 16147 Genoa, Italy; 4 Dipartimento di Neuroscienze, Riabilitazione, Oftalmologia, Genetica e Scienze Materno-Infantili, University of Genoa, 16132 Genoa, Italy

**Keywords:** LC-MS/MS, Adenosine Deaminase, DADA2, enzyme deficiency, autoinflammatory disease

## Abstract

Adenosine Deaminase 2 Deficiency (DADA2) (OMIM: 607575) is a monogenic, autoinflammatory disease caused by the loss of functional homozygous or heterozygous mutations in the ADA 2 gene (previously CECR1, Cat Eye Syndrome Chromosome Region 1). A timely diagnosis is crucial to start Anti-TNF therapies that are efficacious in controlling the disease. The confirmation of DADA2 is based on DNA sequencing and enzymatic assay. It is, thus, very important to have robust and reliable assays that can be rapidly utilized in specialized laboratories that can centralize samples from other centers. In this paper, we show a novel enzymatic assay based on liquid chromatography-tandem mass spectrometry that allows the accurate determination of the ADA2 enzyme activity starting from very small amounts of plasma spotted on filter paper (dried plasma spot). The method allows significantly distinguishing healthy controls from affected patients and carriers and could be of help in implementing the diagnostic workflow of DADA2.

## 1. Introduction

The Deficiency of Adenosine Deaminase 2 (DADA2) (OMIM: 607575) is a monogenic, autoinflammatory disease caused by the loss of functional homozygous or heterozygous mutations in the ADA 2 gene (previously CECR1 Cat Eye Syndrome Chromosome Region 1) [1,2]. DADA2 is characterized by a wide phenotypic and clinical variability. Presentation of the disease includes an early-onset vasculopathy with clinical and histopathological features of polyarteritis nodosa (PAN), associated with hemorrhagic and ischemic strokes. Immunologic and hematologic abnormalities including hypogammaglobulinemia with reduction of memory and terminally differentiated B cells and plasma cells have been described in about half the patients with DADA2 [3]. A severe clinical picture dominated by cytopenia and lymphoproliferation has been also described. Even if the disease’s onset is commonly in the pediatric age, some patients with adulthood onset have been described as well [4]. The clinical course can be chronic or characterized by recurrent flares of systemic inflammation during which the most severe clinical manifestations were described [1].

Several treatments have been used so far in diagnosed patients (steroids, immunosuppressive drugs such as cyclophosphamide, azathioprine, or methotrexate) with the most promising being anti-TNF therapies (etanercept, adalimumab, and infliximab) that have been demonstrated to completely control the inflammatory manifestations preventing the occurrence of vascular events [1].

ADA2 is an isoform of ADA involved in the purine metabolism converting adenosine to inosine and 2′-deoxyadenosine to 2′-deoxyinosine. The other main isoform of ADA is ADA1, whose deficiency is responsible for a severely combined immunodeficiency (SCID). The two isoenzymes present a partial structural homology, but they present some important differences: The affinity of ADA2 for adenosine and deoxyadenosine is about 100 times lower than that of ADA1. ADA1 is monomeric and intracellular, whereas ADA2 is dimeric and it is secreted in the extracellular environment and, therefore, it is detectable in plasma. ADA1 is ubiquitously expressed in all cell types, while ADA2 is predominantly expressed by monocytes and other cells of the myeloid lineage. Temperature and pH stability differ in the two isoforms [5].

A marked reduction of both plasmatic levels and enzymatic activity of ADA2 has been demonstrated in affected patients with respect to healthy donors [6,7]. It has been demonstrated that ADA2 displays a crucial role in the proliferation of monocytes and their differentiation to M2 lineage (anti-inflammatory), thus explaining the pathogenetic role in ADA2 deficiency that is characterized by a prevalence of M1 (pro-inflammatory) cells [6].

Genotyping has detected 18 mutations of ADA2 so far [1], the most frequent being the p.G47R mutation that has been detected in a homozygous state in most patients of Georgian Jewish and Turkish ancestries and the p.R169Q mutation most frequently found in the Caucasian populations living in Northern Europe [6,7]. The frequency of carriers of this mutation in the Georgian Jewish population is 10% [7], whereas in the Caucasian populations in the Northern Europe carriers might be up to two in 1000 individuals [5]. 

It has been demonstrated that a timely diagnosis and treatment is crucial to prevent severe complications of the disease; thus, functional assay is important to confirm the lack of ADA2 activity in suspected patients and represents a rapid tool to support clinicians in starting early treatment with anti-TNF drugs [4].

Current laboratory methods for the determination of ADA2 activity are time consuming and suffer from some limitations that hinder the use of the assays for a rapid screening of patients [4,8,9,10].

In this paper, we describe for the first time a method based on liquid chromatography coupled to tandem mass spectrometry (LC-MS/MS), which allows the fast and reliable determination of ADA2 activity on dried plasma spot (DPS), starting from a very little amount (50 μL) of sample. This method might be of help for a timely diagnosis and a prompt treatment of screened patients.

## 2. Results

### 2.1. UHPLC-MS/MS Method Development and Validation

UHPLC-MS/MS was used to quantify inosine as the main reaction product of ADA2, using adenosine as substrate. Hypoxanthine was used to verify that inosine produced by ADA2 was not consumed by the purine nucleoside phosphorylase (PNP) present in plasma. MS conditions for individual analytes were optimized by direct infusion of standard solutions. Tuning was performed both in positive and in negative ion mode in order to maximize sensitivity. The most intense transition was used as a quantifier, and the second transition was used as qualifier.

Different chromatographic columns and mobile phases (methanol, acetonitrile, and water with or without the addition of 0.1% formic acid) were tested. Optimal peak shape and separation were obtained, using the conditions described in Section 4.9.

Injection of blank samples in triplicate directly after the highest calibration standard showed the absence of carryover for all the analytes. The linear regression fit for the calibration curves was achieved for inosine over the tested range (with an average r2 of 0.97). The results of intra- and inter-assay precision, accuracy, and recoveries were all <15%. The lower limit of quantification (LLOQ) obtained for inosine in DPS was 19.5 ng/mL.

### 2.2. Sample Preparation and Enzyme Reaction

Different sample preparation protocols were tested (as described in Section 4.5). The CV%, calculated on the average of five repetitions, obtained with the six different methods were, respectively, 18.34%, 19.36%, 8.50%, 14,03%, 24.00%, and 19.18%. The scheme able to guarantee a lower CV% was obtained with protocol 3, adding a 5-μL aliquot of adenosine after centrifuging at 2000× *g* for 1 min at 4 °C and 5 min of incubation at 37 °C. Consequently, the described preparation protocol was adopted for further experiments. In order to understand where the exact site of the reaction was, adenosine was let to react in the presence or in the absence of the five 3.2-mm disks obtained from DPS. We found that reaction occurred in the presence of the disks.

The best reaction conditions were obtained using a buffer at pH 6.5 (that is the optimal pH for ADA2 activity) after testing the reaction also in water.

Different extraction protocols were tested. Methanol and 10% formic acid were selected as the extraction solvents after testing different extraction mixtures (methanol, acetonitrile, methanol and zinc sulfate heptahydrate, acetonitrile and zinc sulfate heptahydrate, methanol and trichloroacetic 10%). Different extraction timings (0, 5, 15, and 20 min) were also tested.

The addition of 40 μL of methanol, 10 μL of 10% formic acid, and 15 μL of ammonium bicarbonate 1 M allowed us to stop the reaction and to obtain a good chromatographic separation of analytes.

**Figure 1:**
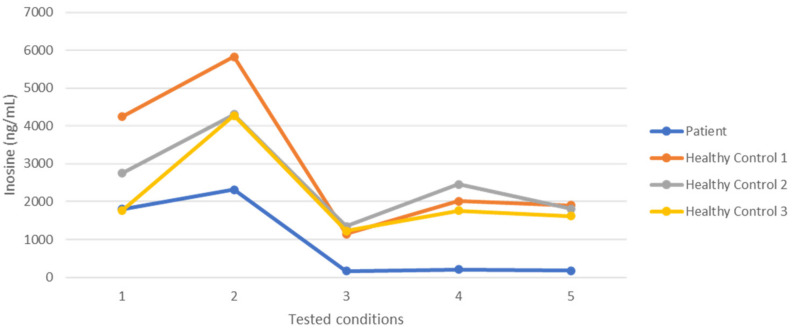
Inosine (ng/mL) produced by a patient and three healthy controls at the following five conditions: EHNA 0 mM and Adenosine 1 mM (1), EHNA 0 mM and Adenosine 2 mM (2), EHNA 1 mM and Adenosine 1 mM (3), EHNA 1 mM and Adenosine 2 mM (4), and EHNA 2 mM and Adenosine 2 mM (5).

The concentrations of erythro-9-Amino-β-hexyl-α-methyl-9H-purine-9-ethanol hydrochloride (EHNA) and Adenosine, described in Section 4.6, were tested on one patient and three controls. Results are shown in Figure 1. The last point of the graph shows the difference obtained between the tested patient and controls. The final concentrations of EHNA and Adenosine were 2 mM.

PNP is involved in the purine metabolism converting inosine to hypoxanthine. The reaction catalyzed by PNP did not occur on DPS; thus; hypoxanthine was not considered for the determination of ADA2 activity. As the basal inosine concentrations resulted to be different in different samples, we decided to consider two time points of the reaction.

### 2.3. ADA2 Activity

ADA2 activity was determined in DPS from 44 healthy donors, 18 DADA2 patients, and four carriers. Patients’ results are shown in Table 1. ADA2 activity, expressed as mean ± SD, was 2.63 ± 1.7 mU/mL in healthy controls, 0.02 ± 0.03 mU/mL in DADA2 patients, and 0.025 ± 0.18 mU/mL in carriers.

**Figure 2:**
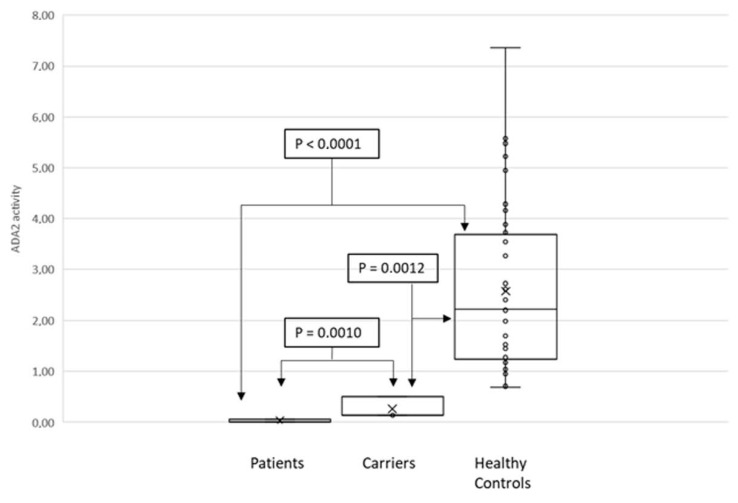
Mann–Whitney tests of ADA2 activity from patients versus carriers (*p* = 0.0010), from patients versus healthy controls (*p* < 0.0010), and from carries versus healthy controls (*p* = 0.0012).

As shown in Figure 2, the Mann–Whitney test showed statistically significant difference (*p* < 0.0001).

### 2.4. Stability

ADA2 activity was stable within 15 days at room temperature (RT), at 4 ± 3 °C, or at −20 °C. The percentage difference, within 20%, could be considered acceptable.

## 3. Discussion

The laboratory diagnosis of ADA2 deficiency is based on genotyping or functional assay on suspected patients. Genotyping includes all nine coding exons (from 2 to 10) of the *ADA2* gene (NM_001282228) analyzed by means of amplification of DNA extracted from peripheral blood lymphocytes followed by direct sequencing. DNA sequencing and enzymatic assay play complementary roles in the diagnosis of DADA2.

Nowadays, anti-TNF therapies are the most promising treatments for the control of the disease, with a dramatic reduction of the probability of a cerebral stroke [4,11]. It has been previously suggested that they should be initiated at the moment of the diagnosis. In this line, it is important to note that the genetic analysis of ADA2 gene could be rather laborious. ADA2 is, in fact, located in the chromosome 22q11, an unstable region subjected to copy number variations and other structural genomic alterations [12]. Therefore, the availability of a fast and reliable functional assay is particularly important in order to alert the clinicians before the results of the genetic analysis in order to start the appropriate treatment as soon as possible.

Very few methods are currently available for the determination of ADA2 enzymatic activity in the literature. The majority of them are based on spectrophotometric assays for quantification of adenosine and inosine [4,8,10] that suffer from lack of specificity if compared to chromatographic methods. An enzymatic assay from circulating monocytes has been previously described by our group [4], which implies the purification of monocytes from fresh peripheral blood and very long incubation times (at least 4 h) at 37 °C before the activity of enzyme can be evaluated. The method was able to distinguish affected patients from healthy controls, detecting ADA2 activity in culture supernatants by HPLC assay. Nevertheless, this test presents some limitations: It requires high volumes of blood (10 mL), it is time consuming including several steps and, most importantly, it does not allow for inter-site shipments, requiring freshly isolated monocytes.

Other authors (Schnappauf Oskar, et al., 2021) [8] described another spectrophotometric assay for the measurement of ADA2 enzymatic activity, quantifying both inosine and hypoxanthine. The assay was compared to a CLIA-certified, HPLC-based method. ADA2 activity (the sum of inosine plus hypoxanthine formed from adenosine during incubation in the presence of EHNA) was expressed as nmols per min (milliunits) per mL of undiluted plasma.

Another recent paper [10] described a colorimetric assay. Interestingly, the authors started from a dried blood spot to determine ADA2 activity, thus allowing easy shipment and storage and limiting the discomfort caused by blood withdrawal. The pitfall of this method is the long incubation time required that represents a limitation for a rapid diagnosis.

To our knowledge, one paper only described an assay based on high-performance liquid chromatography on dried plasma spots on filter paper [9]. The assay was used to confirm ADA deficiency on five patients. The method development and validation are not extensively described and it is based on HPLC with UV-VIS without mass spectrometric detection.

In the present paper, we have shown for the first time the development and validation of a method based on LC-MS/MS for the determination of ADA2 enzyme activity from a dried plasma spot. The use of mass spectrometry as a detection method guarantees a very high specificity. The method is accurate and reproducible, starting from a very low amount (50 μL) of plasma spotted on filter paper. The use of DPS and the possibility of storage and delivery at room temperature allows the analysis of samples collected from remote sites and sent to a reference laboratory in specialized centers. It should be noted that, given the inability in finding an inosine-free plasma, we were not able to use the same matrix for the calibration curve as for the samples. However, since the purpose of our developed method was to verify the enzymatic activity of ADA2 through rapid screening, a slightly inaccurate quantification of inosine could be considered acceptable.

Differently from other authors (Moeko Ito, et al., 2021) [10] who described age-related differences in ADA2 activity, we did not observe differences in enzyme activity between pediatric or adult control subjects. More subjects should be included to draw any conclusion.

Similarly to Schnappauf et al., 2021 [8], our method was able to distinguish carriers from healthy subjects. These findings should be confirmed in a study including a larger population.

In conclusion, in our paper we showed the development of a novel LC-MS/MS method for determining ADA2 activity that is fast (an approximate turnaround time of 3 h from the DPS arrival to the lab to the result), cost effective (as it does not require special reagents), and robust, starting from a very low amount of plasma. Details on method development and validation are provided in order to share protocol details for those interested in reproducing the assay in their laboratory. Moreover, the use of DPS offers the advantage of the inter-site shipment at room temperature allowing the possibility of diagnosing suspected subjects in specialized centers and a rapid diagnosis and treatment start.

## 4. Materials and Methods

### 4.1. Chemicals and Reagents

High-performance liquid chromatography (HPLC)-grade methanol and acetonitrile were purchased from Sigma-Aldrich Srl (Milan, Italy). MS-grade water (MilliQ, manufacture, Milan, Italy) was produced with a Milli-DI system coupled with a Synergy 185 system by Millipore (Milan, Italy). Formic acid (99.9%) was purchased from Merck (Darmstadt, Germany). Trichloroacetic acid, Ammonium bicarbonate, Zinc sulfate heptahydrate, Adenosine, inosine, hypoxanthine, and EHNA were purchased from Sigma Aldrich (Milan, Italy). EHNA hydrochloride purity was >98% while all the other compounds had purity >99%.

### 4.2. Human Samples

Samples were obtained from 18 patients (aged 10–54 years; seven females and 11 males) with ADA2 deficiency confirmed by genotyping and/or functional assay and clinical phenotype, from 44 control subjects (aged 0–96 years; 21 females and 23 males) and from four carriers (aged 49–62; three females and one male). Plasma was obtained from peripheral blood collected in ethylenediaminetetraacetic acid (EDTA)-containing tubes by centrifuging at 2000× *g* for 5 min. Plasma samples were stored at −20 °C until analyzed. A written consent allowing the collection of leftover samples and the use of clinical and nongenetic data for clinical research was signed by patients’ guardians. The study was approved by the Local Ethical Review Board, dated 16 May 2004.

### 4.3. Preparation of Working Solutions, Calibrators, and Quality Control Samples

Stock solutions were prepared by dissolving powdered hypoxanthine in water, powdered adenosine in DMSO, and powdered inosine and ADA1 inhibitor EHNA in buffer (ammonium bicarbonate 10 mM, formic acid 0.02%). Working solutions of each analyte were obtained by diluting the stock solution with appropriate water or buffer volume.

The calibration curve for inosine quantification was composed by 12 points (19, 39, 78, 156, 312, 625, 1250, 2500, 5000, 10,000, 20,000, and 40,000 ng/mL). The four Quality Control (QC) concentrations (LLOQ, QC low, QC medium, and QC high) were: 19.5 ng/mL (LLOQ), 58.5 ng/mL (QC-L), 1000 ng/mL (QC-M), and 30,000 ng/mL (QC-H). Calibrators and QC samples were prepared by diluting the working solution of inosine with appropriate buffer volume. Each calibrator was divided in 50-μL aliquots, and 5 μL of water, 40 μL of methanol, 10 μL of formic acid 10%, and 15 μL of ammonium bicarbonate 1 M were added to each aliquot, in order to mimic changes in volume of patients’ samples. The calibration curves covered the expected concentrations in clinical samples. Linearity was evaluated by analyzing the calibration curve three times on three nonconsecutive days. The acceptance criteria for the variation of the amounts of back-calculated standards were ±15% of the theoretical value (except ±20% for the lowest standard).

### 4.4. Sample Preparation and Enzyme Reaction

A 50-μL aliquot of patients’/controls’ plasma was carefully spotted in duplicate on filter paper using a calibrated pipette and dried at room temperature (20–25 °C) for 2 h. Each DPS was punched to obtain five 3.2-mm-diameter disks (containing approximately 3 μL of plasma) that were put in 1.5-mL Eppendorf tubes.

A 50-μL aliquot of buffer containing 10 mM ammonium bicarbonate, 2 mM EHNA, and 5.9 mM formic acid at pH 6.5 was added. The sample was then centrifuged in an Eppendorf microcentrifuge at 2800× *g* for 1 min at 4 °C and afterwards incubated at 37 °C for 5 min.

The enzyme reaction was carried out in a thermostatic bath at 37 °C adding 5 μL of 22 mM adenosine to obtain a final concentration of adenosine 2 mM. Reactions were then stopped after 5 and 10 min, respectively, by adding 40 μL of methanol and 10 μL of formic acid 10% and vortex mixing each time. After the addition of 15 μL ammonium bicarbonate 1 M, the sample was centrifuged at 20,000× *g* for 5 min at 4 °C. The resulting eluate was directly transferred to auto sampler vials, vortex mixed, and injected in the UHPLC-MS system.

The enzyme activity was generally determined as product formed per time unit. ADA2 activity was expressed as mU/mL. IU is defined as the enzyme amount forming 1 μmol product/min [13].

### 4.5. Analysis of Precision on DPS

Six different sample preparation protocols were tested by the same operator on the same day on the same plasma pool. In each experiment, five 3.2-mm disks were placed in 1.5-mL Eppendorf tubes and 50 μL of buffer were added, as described in Section 2.4.

The tested protocols were the following:

1. A 5-μL aliquot of adenosine was added after 5 min incubation at 37 °C;
2. A 5-μL aliquot of adenosine was added after 10 min incubation at 37 °C;
3. A 5-μL aliquot of adenosine was added after centrifuging at 2000× *g* for 1 min at 4 °C and 5 min of incubation at 37 °C;
4. A 5-μL aliquot of adenosine was added after 10 min incubation at 37 °C and centrifugation at 2000× *g* for 1 min at 4 °C;
5. A 5-μL aliquot of adenosine was added after centrifuging at 14,000× *g* for 1 min at 4 °C and incubation at 37 °C;
6. A 5-μL aliquot of adenosine was added after the sample was sonicated for 5 min at amplitude 10 microns by using a Soniprep 150 (MSE Ltd., London, UK) and then centrifuged for 1 min at 14,000× *g*.

Five duplicates were analyzed for each protocol. The coefficient of variance (CV%) = (SD/mean) 100% was measured for the five repetitions for each protocol.

### 4.6. Study of Kinetic on DPS

The following conditions were tested using plasma from one patient and three controls:

1. EHNA 0 mM and Adenosine 1 mM
2. EHNA 0 mM and Adenosine 2 mM
3. EHNA 1 mM and Adenosine 1 mM
4. EHNA 1 mM and Adenosine 2 mM
5. EHNA 2 mM and Adenosine 2 mM

### 4.7. Within-Run and Between-Run Imprecision

To assess the imprecision [coefficient of variance (CV%) = (SD/mean)·100%] of the ADA2 enzymatic assay, samples from patients and controls were analyzed in triplicates (within-run imprecision). Between-run imprecision experiments were performed on controls (*n* = 16) and by measuring replicates on 3 consecutive days.

### 4.8. Analysis of Stability

Stability was evaluated in triplicate on two different samples, derived from a control and a patient with confirmed ADA2 deficiency, stored at room temperature (25 ± 2 °C) or at 4 ± 3 °C or at −20 °C ± 3 °C in the dark analyzed in triplicate at the following time points: 5, 10, and 15 days.

### 4.9. LC-MS/MS Method Development

LC-MS/MS analyses were performed on a TSQ Quantum Access Max Triple Quadrupole coupled to an Ultimate 3000 UHPLC (Thermofisher Scientific, Milan, Italy). Chromatographic separations were carried out on a Thermofisher Scientific ACQUITY UPLC HSS 1.8 μ.

Gradient separation chromatography was carried out using water (mobile phase A) and methanol (mobile phase B). The percentage of solvent B started at 5% for 1 min, programmed to reach 95% at 1 min and kept for 1 min. Then the column was reconditioned at 5% B, for a total run time of 5 min. The flow rate was 0.4 mL/min. Injection volume was 2 μL and total run time was 5 min. The applied ESI conditions were the following: capillary voltage 3.5 kV (positive polarity) and 2.5 kV (negative polarity), ion transfer tube temperature was 350 °C, and Vaporizer temperature was 300 °C. Nitrogen was used as the nebulizer and auxiliary gas was set at 40 and 10 arbitrary units, respectively. For collision-induced dissociation, high-purity argon was used at a pressure of 1.5 mTorr with a collision energy of 25 V. Q1 and Q3 resolution was 0.7 full-width–half-maximum (FWHM), respectively. Ionization was achieved using electrospray ionization (ESI) in the positive ion mode and analytes were detected using selective reaction monitoring (SRM) of the specific following ion transitions: for adenosine [M + H] 268.194 → 119.317, 136.262, m/z, for inosine [M-H] 267.2 → 135.313 with negative ionization, for hypoxanthine [M + H] 137.089 → 82.278, 92.275, 94.262, 110.231, 119.228.

LC method development was carried out using a standard aqueous stock solution of inosine, adenosine, and hypoxanthine (10 μg/mL). Chromatographic data were collected and analyzed with Xcalibur software. Quantification was achieved for each analyte using quadratic regression analysis of the peak area of inosine (weighed 1/X) versus concentration. Optimization assays were carried out using a standard solution.

Different LC columns were tested during method development and optimization.

### 4.10. Statistical Methods

Comparison of quantitative variables between two groups (patients vs. controls) was made by the Mann–Whitney U test, and among more than two groups (controls vs. carriers and patients) by the nonparametric analysis of variance (Kruskal–Wallis test).

## Figures and Tables

**Table 1:** Characteristics of patients and results of ADA2 activity (expressed in mU/mL).

Patients	Age	Gender	Genotype	Phenotype	ADA2 activity (mU/mL)
Patient 1	22	M	R312X/E328D	vasculitis, livedo reticularis, ipogammaglobulinemia	0.00
Patient 2	30	M	G47R T360A	Fever, livedo reticularis, purpuric lesions, hypertension, stroke, peripheral neurophathy, splenomegaly	0.00
Patient 3	19	M	G47A/L251P	Livedo reticularis recurrent infections	0.05
Patient 4	19	M	L249P/P344L	Livedo reticularis, hypertension, stroke, arthralgia, arthritis	0.05
Patient 5	13	F	Duplication	Hypertension, myocarditis, stroke	0.05
Patient 6	15	M	T360A/T360A	Fever, livedo reticularis, hypertension, stroke, myocarditis	0.00
Patient 7	27	F	S479P/G47V	Livedo reticularis, hypogammaglobulinemia	0.05
Patient 8	25	M	S479P/G47V	Livedo reticularis, hypogammaglobulinemia	0.00
Patient 9	13	M	L188P/G383D	lymphoproliferation, hypogammaglobulinemia	0.04
Patient 10	14	M	R312X/E328D	Livedo reticularis, Stroke, Hypogammaglobulinemia	0.00
Patient 11	20	F	G47R/G47R	PAN, Stroke	0.00
Patient 12	20	F	L180P/T360A	Livedo reticularis, Inflammation, stroke	0.00
Patient 13	15	M	L188P/G383D	lymphoproliferation, hypogammaglobulinemia	0.00
Patient 14	54	M	Y220C omozygous	Polyarteritis nodosa	0.00
Patient 15	10	F	Y453C	Anemia, Livedo reticularis	0.06
Patient 16	36	F	L188P/T187P	Mild Leukopenia, Arthralgia	0.03
Patient 17	31	F	L188P/T187P	PRCA, hypogammaglobulinemia	0.00
Patient 18	26	M	L249P T360A	Systemic hypertension and PRES encephalopathy, livedo Reticularis	0.00

## Data Availability

The data presented in this study are available on request from the corresponding author.
